# Single-Photon Emitting Arrays by Capillary Assembly of Colloidal Semiconductor CdSe/CdS/SiO_2_ Nanocrystals

**DOI:** 10.1021/acsphotonics.3c00351

**Published:** 2023-05-01

**Authors:** Matteo Barelli, Cynthia Vidal, Sergio Fiorito, Alina Myslovska, Dimitrie Cielecki, Vincenzo Aglieri, Iwan Moreels, Riccardo Sapienza, Francesco Di Stasio

**Affiliations:** † Photonic Nanomaterials, Istituto Italiano di Tecnologia, Via Morego 30, 16163 Genoa, Italy; ‡ The Blackett Laboratory, Department of Physics, Imperial College London, London SW7 2AZ, U.K; § Department of Chemistry, Ghent University, 9000 Ghent, Belgium

**Keywords:** quantum dots, chalcogenides, single-photon, nanocrystals, spectroscopy, semiconductors

## Abstract

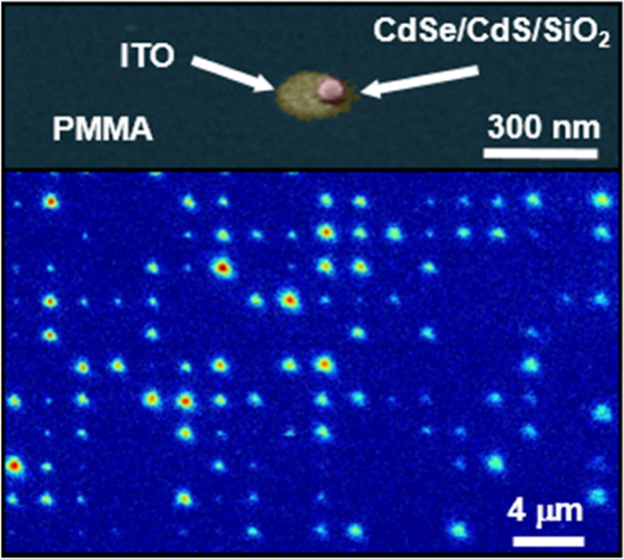

The controlled placement of colloidal semiconductor nanocrystals (NCs) onto planar surfaces is crucial for scalable fabrication of single-photon emitters on-chip, which are critical elements of optical quantum computing, communication, and encryption. The positioning of colloidal semiconductor NCs such as metal chalcogenides or perovskites is still challenging, as it requires a nonaggressive fabrication process to preserve the optical properties of the NCs. In this work, periodic arrays of 2500 nanoholes are patterned by electron beam lithography in a poly(methyl methacrylate) (PMMA) thin film on indium tin oxide/glass substrates. Colloidal core/shell CdSe/CdS NCs, functionalized with a SiO_2_ capping layer to increase their size and facilitate deposition into 100 nm holes, are trapped with a close to optimal Poisson distribution into the PMMA nanoholes via a capillary assembly method. The resulting arrays of NCs contain hundreds of single-photon emitters each. We believe this work paves the way to an affordable, fast, and practical method for the fabrication of nanodevices, such as single-photon-emitting light-emitting diodes based on colloidal semiconductor NCs.

## 1 Introduction

The interest in the physics and materials science of nanocrystals (NCs) has exponentially increased over the last couple of decades thanks to the availability of powerful nanofabrication and characterization tools that greatly improved the performance and affordability of this class of nanomaterials. In particular, research has focused on plasmonic metallic and dielectric nanostructures due to their unparalleled subwavelength light manipulation properties, which are crucial in a wide range of applications such as nanophotonics,^1−3^ sensing,^4,5^ and photocatalysis,^6,7^ and on colloidal semiconductors NCs based on metal chalcogenides and perovskites.^8−10^ Colloidal semiconductor NCs present remarkable properties in sight of optoelectronic device fabrication: tunable, narrow emission line widths and high photoluminescence efficiency for light-emitting diodes,^11,12^ light amplification for lasers,^13^ and desirable light absorption properties for solar cells,^14^ X-ray scintillators, and detectors.^15^ Colloidal semiconductor NCs are synthesized by wet chemistry methods that offer a seemingly endless amount of freedom on the choice of parameters that determine their optoelectronic properties, such as crystal size, shape, crystallinity, chemical composition, and architecture (e.g., core/shell NCs).^8^ Various optoelectronic devices are based on NC thin films;^16^ however, complex small-footprint devices exploiting single NCs require controlled placement of isolated NCs on particular sites and on-chip, beyond what is currently achievable by pure top-down methods. For example, when used as single-photon sources, colloidal semiconductor NCs can power quantum communication, computing, and encryption, where optically pumped quantum dots are quickly gathering attention.^17,18^ In nanophotonics, hybrids of NCs and nanoantennas have been studied extensively, and their operation requires the careful placement of the emitter in the hotspot of the nanoantenna.^19,20^ The integration of colloidal NCs onto single-crystalline semiconductor surfaces is another very technologically relevant topic explored in the literature.^21,22^

Importantly, when in an array, emitters have to be at least an emission wavelength apart to be single-photon sources (and avoid the risk of measuring two sources at the same time with a diffraction-limited laser spot); much more diluted than in a thin compact film. Randomly placed emitters (e.g., NCs deposited from a very diluted solution) are not convenient for practical purposes, they restrain engineering possibilities^23^ and statistically do not guarantee the condition of isolated NCs. A whole range of techniques have been explored to selectively place single colloidal nano- and mesoparticles, especially gold and polymeric colloidal units, from solution to a controlled site on a solid substrate, exploiting, e.g., chemical/wettability contrast between different areas of the substrate,^24^ electrostatic interactions,^25^ optical forces,^26^ scanning probes techniques,^27−30^ and many more.^31^ A method defined as capillary assembly has been used by several groups;^32−34^ yet, such an approach has been only marginally explored with semiconductor NCs.^35−39^ In particular, the capillary assembly technique exploits the force exerted on colloidal particles at the interface between the moving meniscus of the solution solvent and a solid substrate. This allows the entrapment of nanoparticles in prepatterned features, typically realized on the surface by means of lithographic methods such as electron beam lithography.^33,34^ The solution meniscus can be swept on the prepatterned surface by complex instrumentation using a doctor blade-like approach with a slowly moving counter slide (often fractions of μm/s),^37,40,41^ by letting the solution dry on a vertically placed sample^42,43^ or by allowing a droplet of chemically functionalized particles to dry on it.^44^ While the doctor blade-like method is by far the most efficient and documented in the literature, achieving single-particle filling efficiency often near 100% in the case of metal and polymeric nanoparticles, the approach is much less explored for semiconductor NCs, where filling efficiencies, regardless of the method or technique employed, are mostly not clearly reported using a significant statistical analysis, to our knowledge at the time of writing. The controlled placement of colloidal semiconductor NCs remains an outstanding challenge for the fabrication of optoelectronic nanodevices around them.

In this work, we opted for a drop cast capillary assembly approach, since it does not require any particular and costly instrumentation, it is fast, and it allows for efficient use of NCs in solutions. We accurately quantify the achieved filling efficiency over a statistically relevant set of measurements thanks to our large-area arrays. To our knowledge, this is the first demonstration that capillary assembly (albeit without sub-Poissonian efficiency) can be achieved by a simple and fast drop cast of basically unfunctionalized semiconductor colloidal nanoparticles, without any other surface treatment, complex instrumentation, or methods based on delicate electrostatic interactions that may completely quench the NC photoluminescence.^31,37^ The large-area arrays here presented (2500 holes over 100 × 100 μm^2^) each contain hundreds of single NC-filled holes after the colloidal solution drop cast. We have characterized the arrays with steady-state and time-resolved photoluminescence and photon correlation measurements. We correlated morphological data collected by scanning electron microscopy with optical data to unveil how multiple NCs in a nanohole can still act as a single-photon emitter, indicating that a fraction of NCs is not emissive. We believe the proposed technique is the first step toward the realization of affordable and reproducible arrays of single-photon sources based on colloidal semiconductor NCs. Most importantly, the technique enables future implementation of networks of nanodevices, such as single-NC light-emitting diodes, and should be applicable to a wide range of semiconductor NCs with variable compositions and shapes.

## 2 Results and Discussion

**Figure 1:**
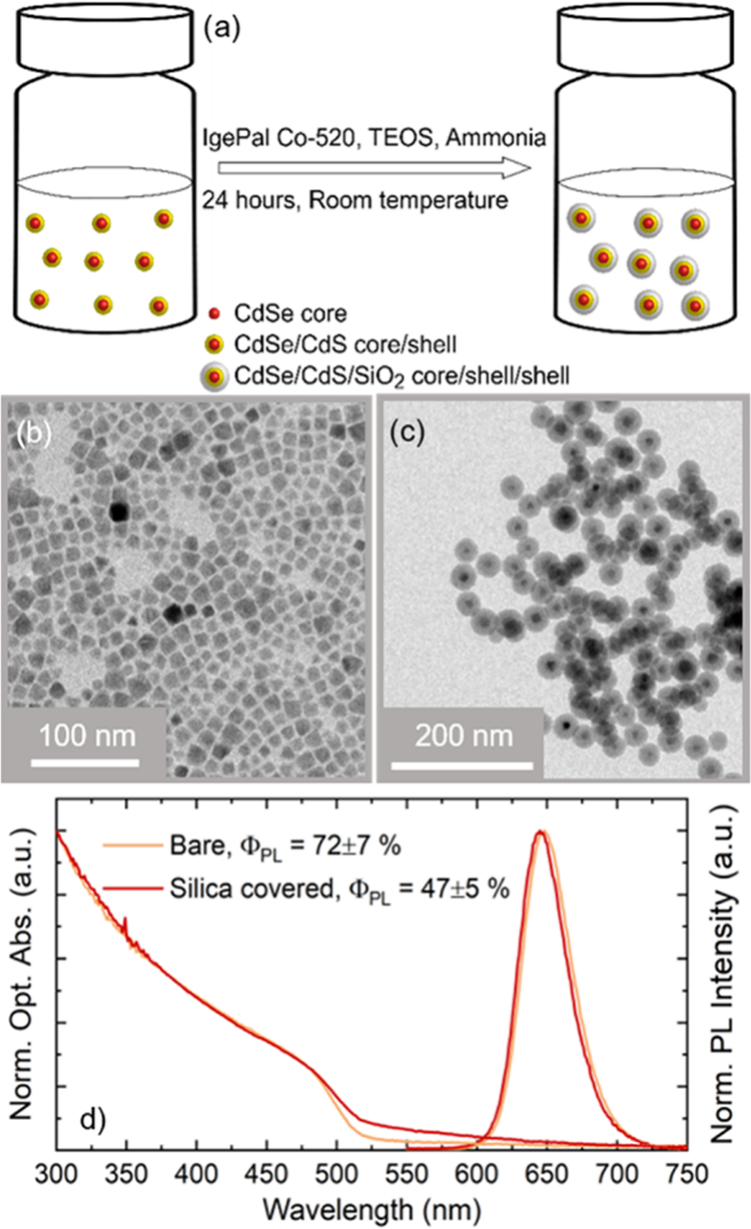
(a) Sketch of the SiO_2_ coating procedure of the giant core–shell CdSe/CdS NCs. TEM images of the NC before (b) and after (c) the SiO_2_ coating process. (d) Optical absorption and photoluminescence spectra of the CdSe/CdS NCs before (bare, light red dashed curve) and after (silica-covered, red solid line) the SiO_2_ coating process.

Giant CdSe/CdS core/shell NCs are synthesized following a protocol reported in the literature with a modification (see the Experimental Section).^45^ Following synthesis, we coated the NCs with a SiO_2_ shell using a previously reported reverse microemulsion procedure (Figure 1a).^46^ The CdSe/CdS NCs have been coated with a SiO_2_ shell to increase the size of the NCs without changing their photoluminescence wavelength. In fact, we need to consider that the typical diameter of holes that are achievable with widely available and standard electron beam lithography (EBL) equipment and common resists is around 100 nm. Employing NCs that are too small compared to the holes’ diameter restrains the probability to have a hole filled with a single NC. Moreover, the SiO_2_ shell stabilizes the NCs in water, as the latter allows deposition of the NCs onto polymer layers commonly employed as resists in electron beam lithography.

The resist layers are produced via spin-coating from nonpolar solvents, and thus, the polymer will dissolve upon exposure to common organic solvents such as toluene or chloroform. In addition, employing water as solvent allows for tuning of the contact angle between a solution droplet and the deposition surface using oxygen plasma or other techniques.^31^ The pristine NCs have a 3.4 nm CdSe core embedded in a shell consisting of 11 CdS monolayers (Figure 1b). Upon SiO_2_ shelling (Figure 1c), the NC diameter increased from 11 ± 5 to 35 ± 3 nm (the reported error is the standard deviation, see Figure S1 in the Supporting Information). Importantly, using optimized SiO_2_ growth parameters (see the Experimental Section), 97% of the silica shells contain CdSe/CdS NCs, while only 3% of them are empty. No silica shells containing more than one NCs were found. Absorption and photoluminescence (PL) spectra of the NCs before and after silica shell growth (Figure 1d) remain nearly unaltered: only a slight blue shift of the PL peak from 648 to 645 nm upon SiO_2_ shelling is observed. On the other hand, the photoluminescence quantum yield (PQLY) of the SiO_2_-coated NCs is reduced to 47 ± 5% (from 72 ± 7%). Such reduction in PLQY is in line with what is reported in the literature^46^ and is ascribed to the involvement of negative charges and hydrolyzed TEOS in the silica coating process.

**Figure 2:**
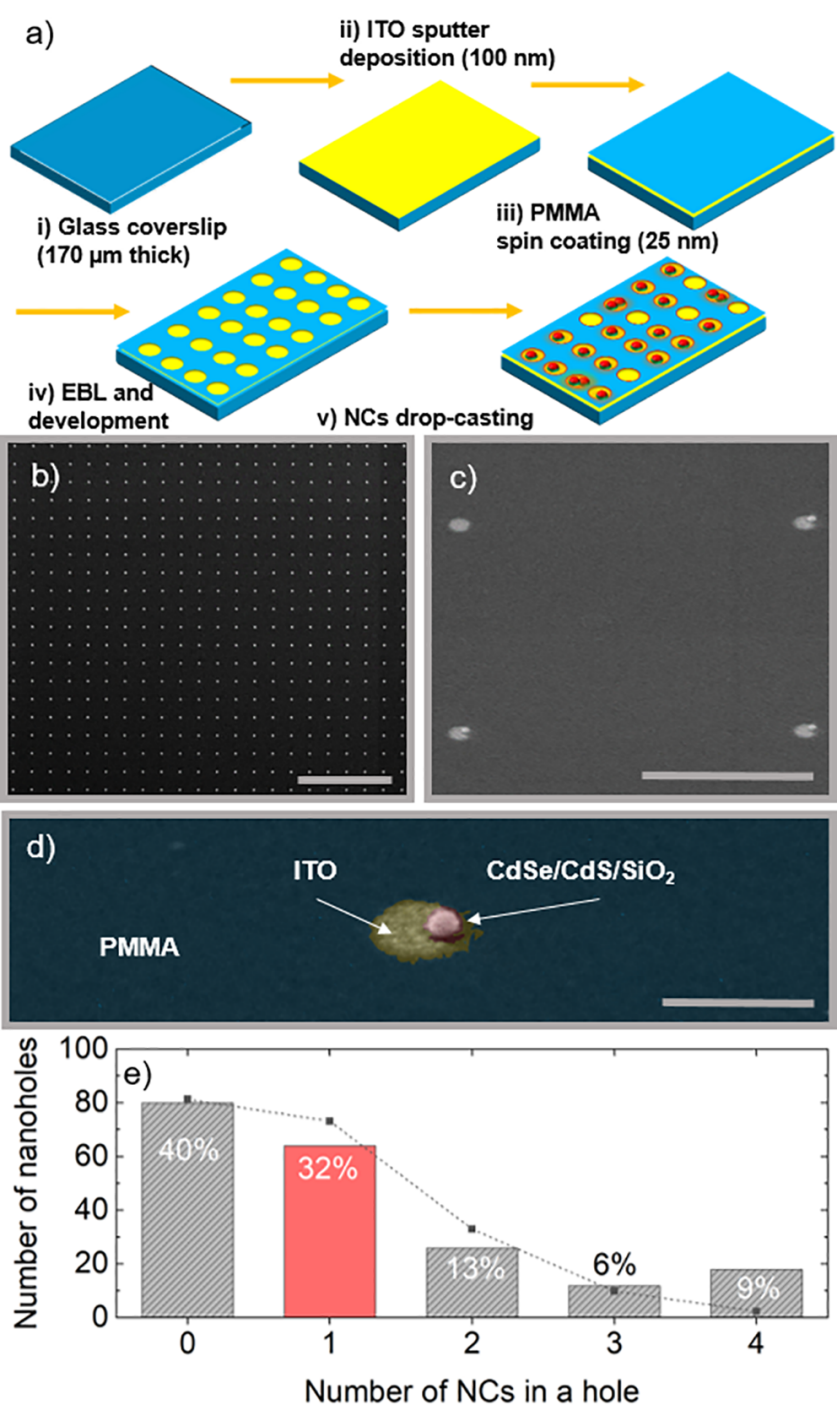
(a) Sketch of the nanopatterned substrate fabrication: (i) 170 μm thick soda-lime glass coverslip. (ii) ITO is deposited on the coverslip (100 nm of thickness) by RF sputtering deposition. (iii) PMMA is spun-coated on the ITO layer. (iv) EBL is performed, exposing several 50 × 50 matrixes of nanoholes. (v) After resist development, the NC solution is drop cast on the sample surface and left to dry as the NCs fill the patterned nanoholes by capillary assembly. (b) Low-resolution scanning electron microscopy (SEM) image of a large portion of one of the developed nanohole arrays. The scale bar is 10 μm. (c) SEM image of a small portion of the nanoholes after the drop cast. There are both empty and filled holes. The surface is clean from stray NCs. The scale bar is 1 μm. (d) High-resolution SEM tilted and colorized image of a nanohole filled with a single NC. The ITO bottom is clearly appreciable; the scale bar is 300 nm. All SEM images are acquired with a secondary electron detector. (e) Nanohole filling efficiency after the drop cast obtained from analysis of an array where 60% of the holes are filled and 32% of the holes contain a single NC (red column). The histogram is built from the analysis of 200 nanoholes. The nanoholes filling follows a Poisson distribution (dashed black line).

Following the synthesis of CdSe/CdS NCs and their functionalization with SiO_2_, we proceeded with the fabrication of the single NCs array. A flat, 18 × 18 mm^2^, 170 μm thick soda-lime glass coverslip (Figure 2a,i) is coated with a 100 nm thick indium tin oxide layer grown by radio frequency (RF) magnetron sputtering (Figure 2a,ii). Afterward, a 25 nm thick poly(methyl methacrylate) (PMMA) film is spin-coated on top of the sample (Figure 2a,iii). Arrays of 100 nm diameter nanoholes are patterned with electron beam lithography (EBL), selectively removing the exposed PMMA (Figure 2a,iv). We typically expose several 100 × 100 μm^2^ arrays, spaced 1 mm from each other. Every array contains a 2500 holes matrix, where each hole has a 2 μm orthogonal periodicity. Each array requires a very short EBL exposure time of about 5 min so that a macroscopical sample can be easily patterned with multiple arrays in a very reasonable time; see the Experimental Section for details. The ITO layer is required for EBL patterning, as it prevents charge buildup during the electron beam exposure and it allows easy observation of the sample after the NCs placement. The nanohole diameter of 100 nm is close to the resolution limit we are able to reliably achieve with our EBL system and development process. Intuitively, as already mentioned when commenting on the growth of the silica shell on the NCs, one would expect that the closer the diameter of the hole to that of the NC, the higher the chance of entrapping only a single NC in it. The thickness of the hole (hence of the resist) was chosen to avoid vertical stacking of the NCs: around 25 nm hole depth versus the 35 nm diameter of the colloidal silica-shelled NCs. The distance of 2 μm between the nanoholes was chosen to guarantee that single NCs in the holes are well above one wavelength of distance between each other, as a necessary condition to observe single-photon emission. The aspect ratio of the holes is indeed important in the capillary assembly process; yet, the issue is not thoroughly investigated in the literature. Some models have been developed for specific processes such as Langmuir Blodgett-based approaches.^38^ Nevertheless, optimal hole aspect ratio values are found as a function of the number of NCs desired in a hole.^35^ Elaborating a model for a much more complex process like the drying of a sessile colloidal droplet is beyond the scope of this work.^47^

Following the substrate preparation, we drop cast a droplet of water-dispersed NCs and let it dry under controlled conditions to entrap the NCs into the prepatterned features (Figure 2a,v). In our experiments, we kept fixed the solid content in the solution used for the drop cast to 0.01%. This concentration is reported in the literature as a reasonable value for metal and polymeric sub-100 nm nanoparticles to favor the creation of an accumulation zone at the meniscus interface that avoids the hindering of the assembly by Brownian motion.^32^ Details on the drop cast method are found in the Experimental Section and Chapter 2 in the Supporting Information (Figures S2, S3, and S4 and Tables S1 and S2).

The quality of our arrays is clearly demonstrated by Figure 2b, which shows that we can easily place NCs only within the nanopatterned holes with the sample surface free from residues and stray NC agglomerates, thus demonstrating precise control on the NC positioning. The holes are visible, as the ITO below shows a strong contrast with the undeveloped dark PMMA surface. In Figure 2c, we show a smaller-area tilted SEM image where one empty nanohole and three filled with single NCs are clearly visible. Finally, in Figure 2d, we show a high-resolution tilted and colorized SEM image of a single nanohole filled with a single NC (see Chapter 3 in the Supporting Information; for additional SEM images, see Figures S5 and S6). Holes can be filled with one or multiple NCs. Figure 2e presents a histogram of the filling efficiency as a function of the number of NCs in a hole built by a statistical analysis of the SEM images. The histogram shows that the nanohole filling follows a Poisson probability distribution (dashed line in Figure 2e), which for a discrete variable *x* is defined as follows
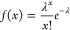
1where λ is a parameter that is equal to the expected value of successful events happening in a given time or space interval and *x* is the number of events that are counted in the relevant interval. The value of λ is also equal to the distribution modal value; therefore, for λ = 1, the probability of filling a hole with a single NC is maximized. The expected value calculated from the Poisson fit of experimental data shows λ = 0.9. The value is remarkably close to the best possible value of λ = 1 for our particular application. From the Poisson fit, we obtain a percentage of nanoholes filled with single NCs as 37%, in close agreement with the sample experimental SEM analysis that reads approximately 32%. Considering that an array contains a 2500 holes matrix, we place about 800 single NCs in a prepatterned site of 100 × 100 μm^2^.

The distribution fit significantly underestimates experimental data for values of 4 or more NCs filling the holes. The reason is that the fit for these numbers has no significant physical value, since we cumulated counts for 4+ NCs instead of considering 4, 5, 6, etc. It is evident from the SEM analysis that when a hole is filled by more than 3 NCs, this leads to partial filling of the hole by a cluster of NCs. The cluster comprehends some NCs residing outside of the hole, and it is difficult to assess the correct number of NCs for each cluster (see Figure S6, the two upper holes to the right). We assign the formation of clusters to the aggregation of NCs, which may take place already in the solution.

The fact that a Poisson distribution applies to our deposition is justified by the following experimental conditions: (i) When the number of possible events greatly exceeds the actual probability of one event actually occurring, a binomial distribution can be approximated by a Poisson one. In our case, the number of NCs drop cast on the surface is much larger than the nanoholes patterned on it, that is, the probability of an NC filling a hole is much smaller than the probability of the NC going elsewhere. (ii) If a NC fills a nanohole, this does not affect the probability of another filling event. That is related to the diameter or the nanoholes being significantly larger than that of the NCs, and this condition holds for several NCs filling the same hole. We may reasonably expect, as we already commented, some significant deviation from a Poisson distribution only in its tails, when NCs are starting to fill a hole, and aggregation from solution becomes an issue. Intuitively, we can expect to improve the probability of filling a nanohole with a NC by decreasing the ratio between the hole and the NC diameters, thus avoiding nanohole filling by multiple NCs, preventing one of the necessary conditions for a Poisson distribution to take place, and intrinsically limiting the filling efficiency. Hence, we can define the array fabrication method as a hybrid approach where we achieve a deterministic spatial positioning of NCs (thanks to the nanohole array) and statistical control over the number of NCs deposited (due to the capillary assembly process).

An individual NC in a nanohole, as observed via SEM analysis, does not guarantee a single-photon source. Instead, validation of the device requires a study of the optical properties at the single-photon level. The second-order autocorrelation function, *g*^(2)^(*t*), of the photoluminescence (PL) originating from individual nanoholes in the array is an unequivocal test of the statistics of the emitted light. The autocorrelation function allows us to determine whether a light source is a single-photon emitter (SPE) by statistically measuring the probability that more than one photon is emitted at the same time (photon bunching), given by *g*^(2)^(*t* = 0). The measurement setup consists of a Hanbury Brown and Twiss interferometer with coincidence counting electronics and avalanche photodiode single-photon detectors (see the Experimental Section). *g*^(2)^(*t* = 0) < 0.5 guarantees that the light source is a SPE, and to recover the value of *g*^(2)^(*t* = 0), the experimental data are fitted as follows^48^
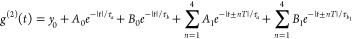
2where *y*_0_, *A*_i_, and τ_j_ are the offset, amplitudes, and lifetimes of the exponential decays, respectively, and *T* = 0.4 μs is the time period of the laser repetition rate. *y*_0_ is the noise baseline specific to each measurement, and the first two terms of the equation correspond to the lifetime contribution of multi-excitons after the first pulsed excitation (*nT* = 0 μs). The last two terms correspond to the lifetime contribution of multi-excitons for the pulsed excitations following the original one (*nT* ≠ 0 μs). Ideally, at low power and with proper filtering, only the exciton decay time, τ_a_, should be measured. However, experimentally and even though exciton decay remains the main photonic process at play, other processes such as biexcitons, trions, and charged states can occur. Their contribution is represented by the decay times, τ_b_, and τ_b1._^45^ We consider that at *n* = 0, the biexciton is the most likely state to occur besides the exciton, whereas at *n* ≠ 0, other multi-exciton states will take place, thereby changing the decay time from τ_b_ to τ_b1_. Here, we make the approximation of representing the lifetime of all of the multi-exciton states with one single average value, which makes sense considering multi-excitons only minimally contribute to the decay rate and is accurate enough for our purpose of retrieving the value of *g*^(2)^(*t* = 0).

**Figure 3:**
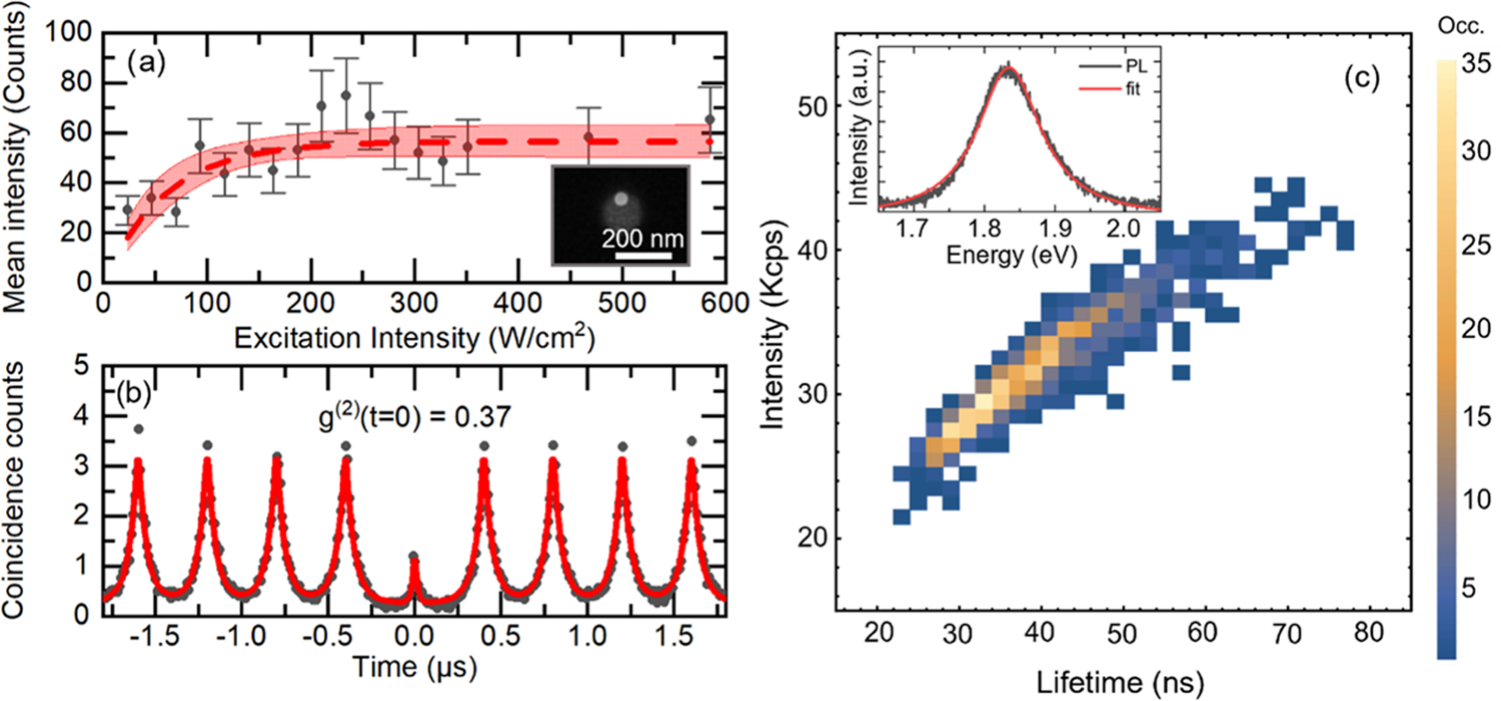
Optical characterization of a single NC at room temperature. (a) Saturation curve of the NC presenting a saturation intensity *I*_s_ of 54 W/cm^2^. All the following measurements are carried out below saturation at *I* = 47 W/cm^2^. Inset in panel a: SEM image of the single NC measured in panels a, b, and c. (b) Autocorrelation curve of the NC with *g*^(2)^(*t* = 0) = 0.37, confirming the NC acts as a SPE. (c) FLID shows the presence of a main state with average lifetime τ_1_ = 30 ns and *I*_1_ = 30 kcps, corresponding to the SPE state measured in b. Inset in panel c: PL of the single NC measured in panels a, b, and c, corresponding Lorentzian fit. The fitted (red curve) PL spectrum (black curve) confirms that the emission corresponds to a single two-level system, most likely the exciton.

In Figure 3, we present the optical characterization of a single CdSe/CdS/SiO_2_ NC deposited in a nanohole. The SEM image (inset in Figure 3a) shows the presence of a single NC in the nanohole. We measure its saturation intensity, autocorrelation function, *g*^(2)^(*t*), and fluorescence lifetime intensity distribution in Figure 3a–c, respectively (see Figure S7 for additional data). After fitting (red dashed curve in Figure 3a), we determine the saturation intensity of the NC to be *P*_s_ = 54 W/cm^2^ (see refined measurement for saturation intensity in Figure S8). All of the following measurements were carried out at *P* = 47 W/cm^2^, i.e., below saturation. The value of the autocorrelation measurements, *g*^(2)^(*t* = 0) = 0.37 < 0.5 (Figure 3b), confirms that the NC is a SPE. The fluorescence lifetime intensity distribution, FLID (Figure 3c), allows the discrimination of the different emission states from the NC. The PL emission intensity and lifetime are measured simultaneously for 180 s. The data analysis consists of fitting the lifetime with a single exponential for time intervals of milliseconds (see the Experimental Section for a more detailed explanation of FLID data analysis). The FLID is built by plotting the density of occurrences with a specific lifetime and intensity, thus resolving the various emitting states of the NC. Here, the FLID displays one main state with average lifetime τ_1_ = 30 ns and intensity *I*_1_ = 30 kcps tailing toward longer brighter states. The SPE originates from the main state displayed here, which corresponds to single-exciton emission confirmed by the Lorentzian fit of the PL spectrum in the inset of Figure 3c.

**Figure 4:**
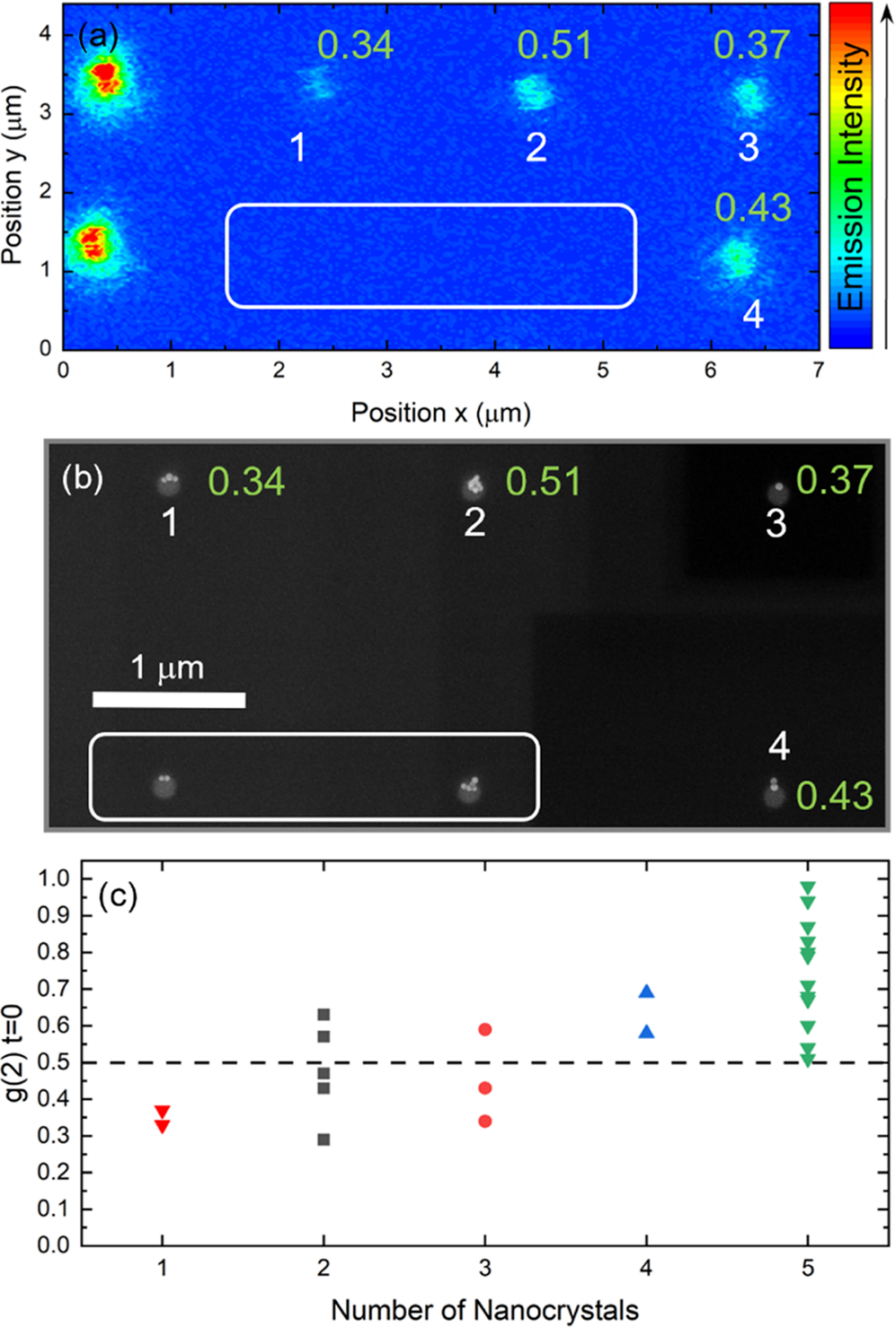
Correlative microscopy of confocal and SEM images with autocorrelation measurements. (a) Confocal PL image of the nanoholes with (b) its corresponding SEM image. Scale bar: 1 μm. The nanohole numbering is indicated in white underneath it and the corresponding *g*^(2)^(*t* = 0) value in gray next to it in panel a and in green in panel b. (c) *g*^(2)^(*t* = 0) values as a function of the number of NCs per nanohole determined from the SEM images. The nanoholes containing 5 or more NCs are included in column "5 NCs".

Figure 4 shows the correlative microscopy of the scanning confocal image (Figure 4a), the SEM image (Figure 4b), and *g*^(2)^(*t*) measurement of the nanohole array (Figure 4c). Nanoholes in the array can be addressed individually to determine the value of *g*^(2)^(*t* = 0) (indicated in green in Figure 4a,b).

The comparative analysis of SEM images and *g*^(2)^(*t* = 0) shows that SPEs can arise from nanoholes containing more than a single NC, as illustrated in Figure 4b, by nanoholes #1, #3, and # 4. As expected by the nature of our experiment (excitation below saturation, filtering of multi-exciton contributions), nanoholes containing a single NC as #3 act as an SPE. However, more unexpectedly, nanoholes containing more than a single NC such as #1 and #4 in Figure 4b also act as SPEs with *g*^(2)^(*t* = 0) values of 0.34 and 0.43, respectively. Figure 4c plots the value of *g*^(2)^(*t* = 0) as a function of the number of NCs per nanohole (the complete set of measurements is reported in Table S3 for clarity). For example, nanoholes containing up to 3 NCs can still act as SPEs; however, there is no correlation between the number of NCs in a nanohole and the value of *g*^(2)^(*t* = 0). This observation can be explained by considering that a fraction of NCs do not emit light. The presence of a nonemissive fraction of NCs is displayed in the comparative SEM and optical images in Figure 4a,b (white frames). Finally, yet importantly, Lorentzian fits of emission spectra of nanoholes #1 and #3 (Figure S9) confirm the single-state emission nature of these SPEs in spite of the number of NCs in the hole. The nonemitting NCs could already be present as such in solution before deposition, or the deposition process at 45 °C may induce some damage (i.e., ligand detachment) and reduction of the emission of the NCs. It is however clear that those NCs that act as SPEs also retain the overall optical PL properties of giant-shell semiconductor NCs as shown by the comparison between NCs before and after silica coating in Figures 1 and S10 as well as in previous studies of giant-shell NCs.^49^ These measurements quantitatively correlate imaging and single-photon emission properties of semiconductor NCs. They demonstrate that in principle, the measured *g*^(2)^(*t* = 0) values below 0.5 do not necessarily imply the physical presence of a single NC under the excitation beam when the PLQY of NCs does not reach unity. These evaluations were enabled by the very large number of NCs confined in easily selectable positions in our large-area nanohole arrays (2500 nanoholes in a single array), making the extraction of significant statistical information possible.

## 3 Conclusions

In summary, we have demonstrated a simple yet powerful method for the fabrication of single NC arrays, which enables spatial positioning of hundreds of single NCs acting as SPEs. Our process is based on the interplay between top-down (electron beam lithography) and bottom-up (capillary assembly) fabrication and, it is a hybrid approach that is compatible with a wide range of NCs and EBL systems, as it employs relatively large nanoholes (100 nm in diameter) and a widely applicable surface functionalization of the NCs (overcoating with a SiO_2_ shell). As a proof of concept, we have demonstrated that our arrays enable correlative optical/electron microscopy measurements thanks to the positioning of NCs in the nanoholes. Furthermore, the extensive number of deposited NCs allows us to carry out a thorough investigation of the NCs with statistical analysis of the emission properties, which matches the predictions of a Poisson distribution of NCs in the nanoholes arrays.

The deposition must be further improved to reach higher spatial accuracy and larger array sizes, toward deposition on metasurfaces for nanophotonic engineering, as well as electrical circuitry for optoelectronic integration, opening a path toward multiplexed on-chip single-photon sources for quantum technologies. We demonstrated that we can control the positioning of NCs; yet, the statistical filling of the patterned holes with NCs requires further improvement. For example, employing NCs with large diameters matching the hole size and imparting chemical affinity between the NC surface and the exposed ITO in the holes are possible routes to push the filling with a single NC toward 100%. Importantly, while this manuscript was under review, a paper reporting a very similar approach to ours has been published.^50^

## 4 Experimental Section

### 4.1 Giant-Shell CdSe/CdS Nanocrystal Synthesis

#### 4.1.1 Materials

Cadmium oxide (CdO, 99. 99%, Aldrich), n-octadecylphosphonic acid (ODPA, 97%, Plasma Chem), tetradecylphosphonic acid (TDPA, 97%, Plasma Chem), trioctylphosphine oxide (TOPO, Merck), trioctylphosphine (TOP, 97%, Strem Chemicals), selenium powder (Se, 99. 5%, Aldrich), and solvents: hexane, toluene, and methanol (ChemLab Analytical) were used in the paper.

#### 4.1.2 Synthesis of the Sulfur Precursor (TOP-S)

A solution of 0.5 M TOP-S was prepared by dissolving 512 mg of S in 16 mL of TOP and 16 mL of ODE under an inert atmosphere at 120 °C for 20 min.

#### 4.1.3 Synthesis of 0.5 M Cd(ol)_2_

2.568 g of CdO (20.06 mmol), 20 mL of oleic acid, and 20 mL of ODE were added to a 50 mL three-necked flask. The mixture was degassed at 120 °C for 30 min. Degassing was followed by heating the mixture to 280 °C under an inert atmosphere for CdO to dissolve and then left for 10–15 min at 210 °C to obtain a colorless solution. Then, the reaction mixture was again degassed for 30 min at 120 °C.

#### 4.1.4 Synthesis of Colloidal CdSe/CdS Core/Shell Nanocrystals

3.4 nm CdSe core-only nanocrystals were synthesized analogous to the procedure in ref (38). The CdS shell growth procedure was performed in accordance with ref (39). A 50 mL three-neck flask was loaded with 60 nmol of previously synthesized CdSe cores, 500 μL of a 0.5 M Cd(ol)_2_ solution, and 10 mL of ODE. The solution was degassed for 20 min at 100 °C and subsequently heated up to 280 °C. Meanwhile, in a separate vial, 2 mL of 0.5 M TOP-S, 2 mL of ODE, 1 mL of TOP, 2 mL of 0.5 M Cd(ol)_2_, and 1 mL of oleic acid were mixed. This solution was then injected into the solution comprising CdSe cores in the flask under nitrogen flow at a rate of 2 mL per hour by means of a syringe pump. When the injection was completed, the reaction was left to cool down to room temperature. 25 mL of isopropanol were added in order to precipitate the quantum dots. The quantum dots were then centrifuged for 10 min at 5000 rpm. The supernatant was discarded and the precipitate was redispersed in hexane.

### 4.2 Silica Coating of CdSe/CdS Nanocrystals

For the silica coating of giant-shell nanocrystals, 1.3 mL of IgePAL CO-520 were dispersed in 10 mL of cyclohexane and stirred in a 20 mL glass vial for 15 min at 850 rpm. Afterward, 0.5 mL of a hexane dispersion of CdSe/CdS giant-shell nanocrystals (≈1 nmol) and 80 μL of tetraethyl orthosilicate were added to the mixture, each addition followed by a new stirring step. Silica growth was finally triggered by the addition of 150 μL of ammonia solution and the reaction was carried out at room temperature and in the dark for 21 h. The washing of the samples from the reaction batch and unreacted precursors was then accomplished through three subsequent ethanol addition (3, 10, and 10 mL), each followed by a different centrifuging step (10, 20, and 40 min at 6000 rcf).^40^

The final concentration of silica-shelled NCs was measured by ICP spectroscopy to be about 1 μM. The solution solid content can be easily estimated by dividing a unit of volume by the volume of NCs therein contained (using the average NC diameter found by TEM analysis and idealizing them as perfect spheres and starting solution molarity). For experiments, we diluted the solution in water using an NC solid content value of about 0.01% in the droplets cast on the sample.

### 4.3 Nanopatterned Substrate Fabrication

A thin (170 μm) soda-lime glass coverslip (18 × 18 mm^2^) was rinsed sequentially with acetone, isopropanol, and distilled water. A thin film of ITO (100 nm) was grown on the glass coverslip by radio frequency (RF) magnetron sputtering of an ITO target by means of a Kenosistec KS500 confocal sputter coater system, achieving an RMS surface roughness of about 1 nm. The process was performed at room temperature, while water-cooling the sample holder, at an RF power of 80 W, an argon flux of 25 sccm, an O_2_ flux of 0.5 sccm, and a total pressure of 3 μbar (a base vacuum of 2 × 10^–6^ mbar). The samples were again gently rinsed with isopropanol and water, followed by ITO deposition. When optimal surface cleanliness was reached, an anisole 1:1 diluted solution of PMMA A2 was spun-coated at 3000 rpm on the sample, achieving a film thickness of about 20–25 nm. Electron beam lithography (EBL) was then performed, patterning matrixes of nanoholes of about 100 nm diameter, spaced by 2 μm from each other both vertically and horizontally. The EBL system used was a Raith150 Two, the beam energy was set to 20 kV, and the beam current was about 30 pA. Finally, the electron-exposed PMMA resist was developed in a 1:3 MIBK/IPA solution for 30 s, and the development was stopped after rinsing for another 30 sec in IPA.

### 4.4 Drop Cast Process

8 μL of a solution of NCs (consisting of a mixture of water and ethanol (85–15% in volume)) was drop cast on the nanopatterned areas of the sample and left to dry at 45 °C. The solid content percentage was fixed at 0.01% (see Silica Coating of CdSe/CdS Nanocrystals in the Experimental Section). For a detailed description of the drop cast optimization process, which led to the choice of these parameters, please refer to the Supporting Information paragraph 2 “Colloidal drop cast and capillary assembly process discussion”. In this case, the contact angle between the colloidal droplet and the solid substrate read an average value of 52.2 ± 0.8°. We avoided the use of oxygen plasma to lower the contact angle because of the etching effect it had on the PMMA and the scarce control, homogeneity over the sample, and reproducibility of the resulting water/PMMA contact angle. Instead, we explored the tailoring of the contact angle by adding ethanol to the colloidal solution to achieve a smoother and nondestructive tuning (see the Supporting Information). Contact angle measurements were performed by dropping five 8 μL droplets along different coordinates of each measured sample and acquiring the values of right and left contact angles. The instrument used to perform the measurements was an OCAH-200 Dataphysics, and the analysis software was Dataphysics SCA22.

### 4.5 Morphological Characterization

Transmission electron microscopy (TEM) images were acquired on a JEOL JEM-1011 microscope equipped with a thermionic gun at 100 kV accelerating voltage. The samples were prepared by drop-casting diluted nanocrystal suspensions onto 200-mesh carbon-coated copper grids. Top view and tilted scanning electron microscopy images were acquired with an FEI Helios Nanolab 650 using the secondary electron channel, a beam energy of 2 kV, and a beam current of 50 pA.

### 4.6 Optical Characterization

Photoluminescence (PL) measurements carried out on individual emitters were performed with a 440 nm, 80 ps-pulsed laser at a repetition rate of 2.5 MHz, through a confocal microscope setup operated in reflection mode with a 100x, NA1.45 objective lens. Antibunching measurements were carried out using a Hanbury Brown and Twiss (HBT) interferometer with two avalanche photodiodes (APDs) and a PicoQuant Time-Correlated Single Photon Counter (TCSPC). The PL emission from NCs was filtered in detection using a band-pass filter centered on 650 nm, corresponding to the PL peak of the NCs and a short-pass filter at 700 nm to remove any afterglow from the APDs in the TCSPC signal. The emitters were first pinpointed by scanning confocal imaging using the signal from one APD, and then antibunching measurements were performed for 180 s at a resolution of 25 ps per time bin on each emitter present in the image to build the statistics.

The lifetime decay histogram of a single emitter consists of multiple exponentials, each corresponding to a different excitonic state (e.g., neutral excitons, biexcitons, trions). In order to determine the lifetime of the neutral exciton and assess the contribution of other states, we performed measurements that allow us to generate a fluorescence lifetime intensity distribution (FLID). To perform data analysis for FLID, we assume that for short time windows, the NCs emit from a single state, with possible small contributions from charging, before blinking occurs. Therefore, we can split the stream of photon arrival times into equal time intervals of 100 ms corresponding to the longest lifetime, which can be fitted as a single exponential. The lifetime–intensity pairs are retrieved for all of the time intervals and plotted as a density distribution in Figure 3c. PL and PLQY measurements in solution were performed using an Edinburgh Instruments fluorescence spectrometer (FLS920) equipped with a xenon lamp with a monochromator for steady-state PL (λ_exc_ = 400 nm) and a pulsed laser for time-resolved measurements (λ_exc_ = 400 nm). A calibrated integrating sphere was used to record the PLQY values from diluted solutions.

## Supporting Information Available

Additional morphological data and analysis; colloidal suspension drop cast and capillary assembly process discussion; additional SEM images of the nanohole arrays; optical characterization of a single NC; and mathematical model (PDF)
