# Supplementary material for: Single-Photon Emitting Arrays by Capillary Assembly of Colloidal Semiconductor CdSe/CdS/SiO2 Nanocrystals
Source: ACS Photonics. 2023 May 1;10(5):1662–70. doi: 10.1021/acsphotonics.3c00351 (PMC10197167; doi:10.1021/acsphotonics.3c00351)
Supplement: Supplementary file 1 — ph3c00351_si_001.pdf [file ph3c00351_si_001.pdf]

# SUPPORTING INFORMATION

**Single Photon Emitting Arrays by Capillary Assembly of Colloidal Semiconductor $CdSe/CdS/SiO_2$ Nanocrystals**

*Matteo Barelli[1†], Cynthia Vidal[2†], Sergio Fiorito[1], Alina Myslovska[3], Dimitrie Cielecki[3], Vincenzo Aglieri[1], Iwan Moreels[3*], Riccardo Sapienza[2*] and Francesco Di Stasio[1*]*

[1] Photonic Nanomaterials, Istituto Italiano di Tecnologia, Via Morego 30, 16163, Genoa, Italy

[2] The Blackett Laboratory, Department of Physics, Imperial College London, London SW7 2AZ, U.K.

[3] Department of Chemistry, Ghent University, 9000 Ghent, Belgium

* To whom correspondence may be addressed:

Iwan.Moreels@UGent.be, R.Sapienza@imperial.ac.uk, Francesco.Distasio@iit.it

† Matteo Barelli and Cynthia Vidal contributed equally to this work.

## 1. Additional morphological data and analysis

In Figure S1 we show the size histograms of bare CdSe/Cds NCs (pink bars) and CdSe/Cds covered by the $SiO_2$ shell (red bars). Upon $SiO_2$ shelling, the average NC size increases from 11±5 nm to 35±3 nm.

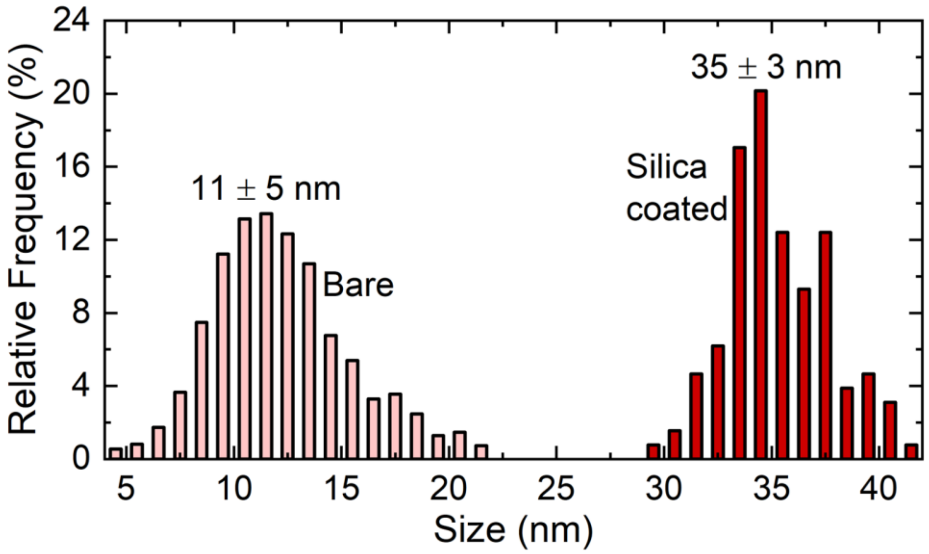


**Figure S1** – *Size histograms of bare CdSe/Cds NCs (pink bars) and CdSe/Cds covered by the $SiO_2$ shell (red bars).*

## 2. Colloidal suspension dropcast and capillary assembly process discussion

In Figure S2 we show a basic sketch of the principle of capillary assembly. The evaporation of the solvent of the colloidal droplet drags the particle to the meniscus due to a chemical gradient and subsequent convective solvent flow created by a faster evaporation rate at the border of the droplet. At the meniscus interface with air and the substrate, the colloidal particles experience two main forces: i) a force parallel to the substrate that drags the particles towards the center of the droplet (due to the diminishing size of the droplet upon evaporation) and ii) a normal force with respect to the substrate which pushes particles against the solid surface. The balance between these two forces is a function of the colloidal solution contact angle (CA) with the substrate. Its value is critical for a successful capillary assembly (putting the particles inside pre-patterned features) or convective assembly (creating monolayers or 2D structures).[1–3] There are more complex particles flows inside the droplet (such as Marangoni flows) and many more relevant parameters for the

control of the process but this discussion goes beyond the scope of this work. In the main manuscript, we addressed relevant literature that the interested reader can refer to.[4,5]

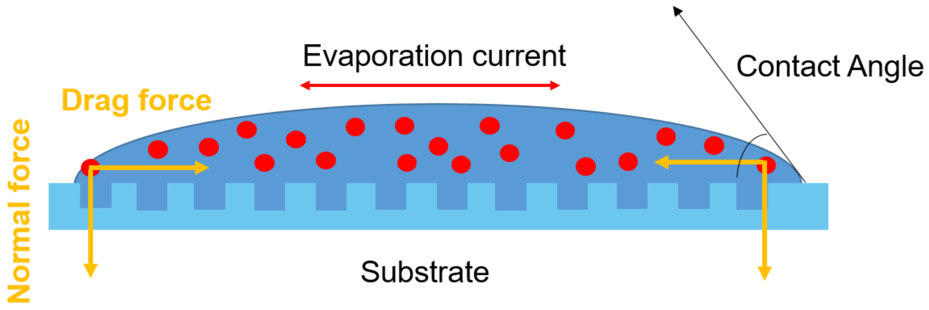


**Figure S2** – *Simple sketch of forces and particle flows involved in the drying of a droplet of colloidal solution.*

In our experiments, we kept fixed the solid content inside the solution used for the dropcast to about 0.01 %, into a range that is reported in literature as a reasonable value for metal and polymeric sub-100 nm nanoparticles to favor the creation of a nanoparticles accumulation zone at the meniscus interface that avoids the hindering of the assembly by Brownian motion.[3] Preliminary optimization studies led us to choose a drying temperature of 45 °C to avoid coffee ring effects appearing at lower temperatures, thus preventing particles to confine and localize themselves on the outer border of the drying droplet, outside the hole patterned areas.[4,5] Higher temperatures led to an excessively fast meniscus drying speed, faster than reasonable ranges reported in literature and to chaotic dried patterns. We focused then on the exploration of the CA experimental parameter to optimize as much as possible the NCs hole filling efficiency.

| [redacted] | RCA (°) | LCA (°) |
|---|---|---|
| Untreated PMMA | 70 ± 1 | 70 ± 1 |
| $O_2$ plasma (100 W – 10 s) | 42 ± 5 | 42 ± 5 |

**Table S1** – *Right contact angle (RCA) and left contact angle (LCA) average values for untreated PMMA and for PMMA after $O_2$ plasma treatment.*

Untreated PMMA has a very high CA with water of about 70° (Fig. S3a and Table S1, see the main manuscript experimental section for the measurements details). The presence of the ITO thin film made no difference on the measured values compared to a sample with PMMA spun directly on a glass substrate (data not shown). After the dropcast process we observe that the drag force

largely dominates and capillary assembly is not achieved (Fig. S3c). The droplet dries into a small concentrated dot, a well-known dried colloidal drop shape in literature when the solution-surface interaction is hydrophobic. By lowering the CA with a plasma treatment to about 42° (Table S1, Figure S3b) the droplet dries over a much larger, coffee stain free area. Some holes are filled with NCs (also single NCs) but we observed very poor filling efficiency, and multiple stray NCs and agglomerates outside the holes are present (Fig. S3d) because of an excess of normal force. The standard deviation of the $O_2$ treated PMMA CAs is very high (Table S1); the treatment is not homogenous over the sample surface and often quite asymmetric droplets are found. Moreover, this CA value is not very tunable with $O_2$ plasma treatment and time, and it etches the PMMA resist leading to even more scarce overall reproducibility of the experiments.

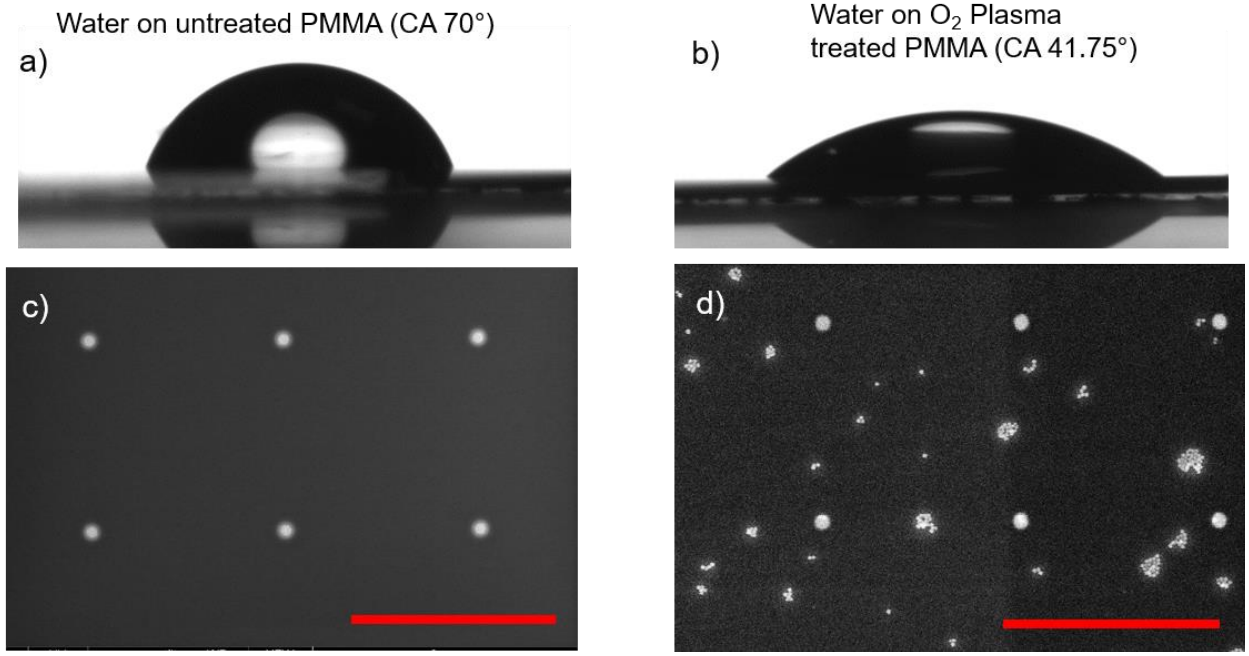


**Figure S3** – *a) Picture of a 8 uL water droplet onto untreated PMMA surface and b) onto $O_2$ treated PMMA surface. c) SEM image of a small area of an EBL patterned hole array after the dropcast process on the untreated PMMA surface and d) on the $O_2$ plasma treated PMMA. Scale bars on the SEM images are 2 um.*

We then opted to tune the CA by adding a varying ethanol volume percentage to the NC aqueous solution. As shown in Table S2 the average CA tends to get lower as the ethanol percentage is increased, allowing for a finer tuning of this parameter in a nondestructive and more homogenous way over the sample, evidenced by the much lower standard deviation compared to the $O_2$ plasma treatment case (calculated form 5 different samples). CA values for 10, 15 and 20% of ethanol

volume are quite similar and we achieved indeed similar NCs filling efficiencies (those reported in the main manuscript) approaching a Poisson distribution, the maximum efficiency we can achieve with holes diameter significantly bigger than the NCs. For 5% of ethanol amount we did not observe any capillary assembly (drag force dominant) and with the highest amount of 25% we again observed a low filling efficiency and many stray nanocrystals and agglomerates (normal force dominant).

| Ethanol (%) | RCA (°) | LCA (°) |
|---|---|---|
| 0 | 70 ± 1 | 70 ± 1 |
| 5 | 62 ± 2 | 62 ± 3 |
| 10 | 53 ± 1 | 55 ± 1 |
| 15 | 52 ± 1 | 52 ± 0.5 |
| 20 | 51 ± 4 | 51 ± 2 |
| 25 | 46 ± 1 | 46 ± 1 |

**Table S2** – *Right contact angle (RCA) and left contact angle (LCA) average values for NCs aqueous solution varying volume percentage of ethanol content.*

The presence of ethanol surely complicates and possibly increases the phenomena behind the droplet drying process (e.g. changing the Marangoni flows inside the drying solution), but it certainly proved to be a versatile way to tune the crucial CA parameter and achieve a capillary assembly with almost maximum efficiency allowed by the nanoholes size: a Poisson distribution. In Fig. S4a,d we show how adding 5% ethanol leads to results similar to pure water where the CA is too high and no NC assembly takes place. In Fig. S4b,e we show how, by lowering the CA via addition of 15% of ethanol to the water solution, the normal force exerted on the NCs on the droplet is able to trap them in the nanoholes. The drag force is still sufficient to have a clean surface. In Fig. S4c,f instead the amount of ethanol of 25% is too high, normal force dominates leading to the random deposition of 2D assemblies of NCs.

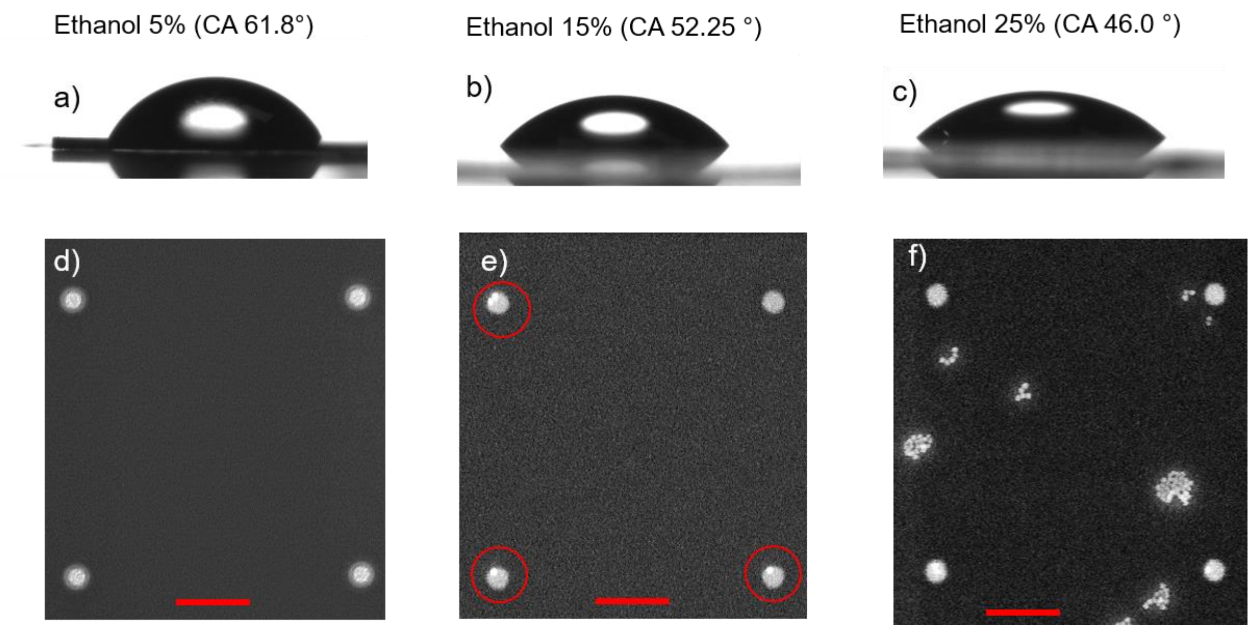


**Figure S4** – *a, b, c) Pictures of 8 uL NC droplets for a 5, 15 and 25 % ethanol contents, respectively. d, e, f) SEM images of a detail of EBL patterned holes arrays after NC solution dropcast with 5, 15 and 25 % ethanol contents, respectively. Scale bars are 300 nm.*

## 3. Additional SEM images of the nanoholes arrays

In Figures S5 and S6 we show additional SEM images of the NCs filled nanoholes array. Figure S6 shows how filling by multiple NCs can lead to clusters only partially filling the holes.

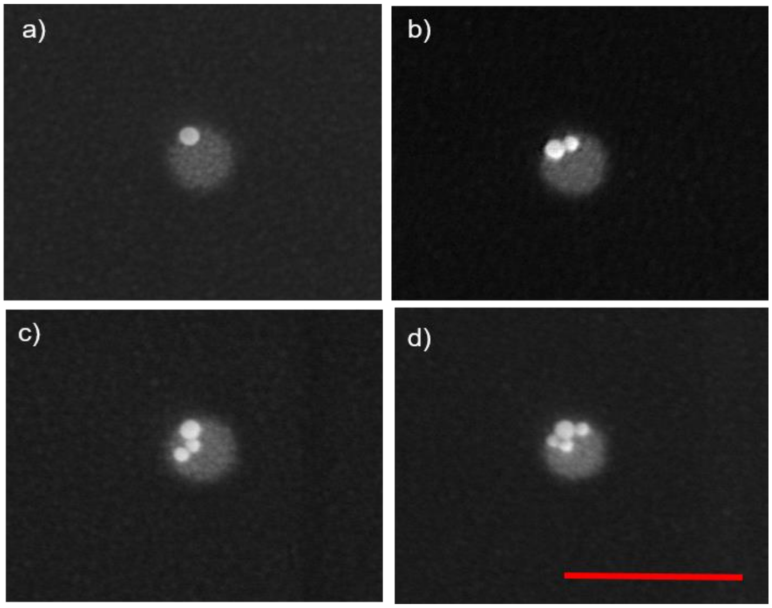


**Figure S5 –** *SEM image of a nanohole filled with a) one b) two c) three and d) four NCs. The red scale bar in panel d) is 400 nm and is shared with all the other panels of the figure.*

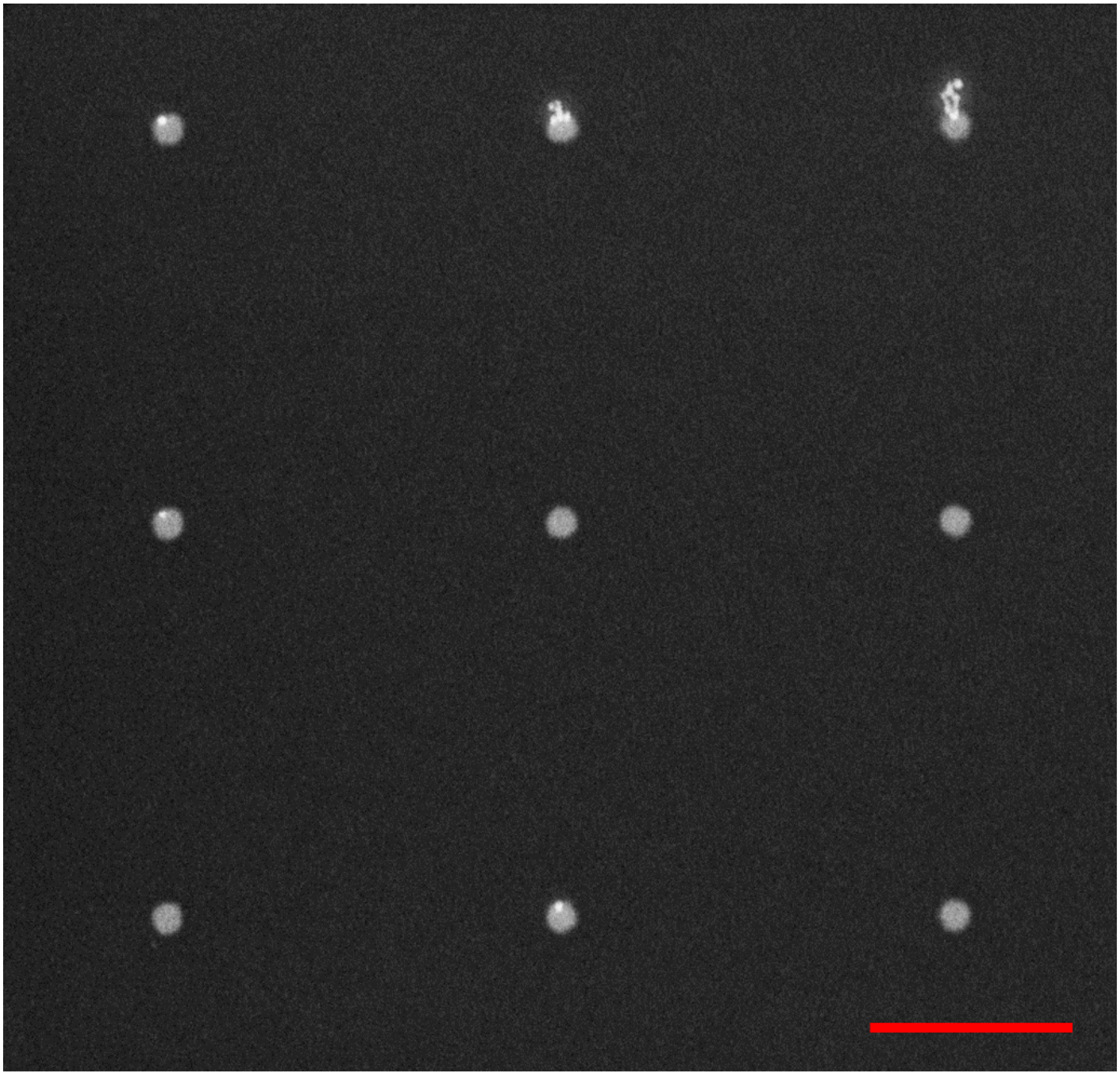

**Figure S6** – *SEM image of a detail of nanoholes array after NCs dropcast. The red scale bar is 1 µm.*

## 4. Optical characterization of a single NC

The intensity-time trace in Figure S7a shows flickering as opposed to telegraphic blinking observed in CdSe/CdS core/shell NCs, which may be the result of the additional $SiO_2$ shell. The lifetime measurement presents a double exponential decay with lifetime values of $\tau_1$ = 30 ns and $\tau_2$ = 61 ns (Figure S7b). These lifetimes are consistent with the emission of an exciton and a biexciton.

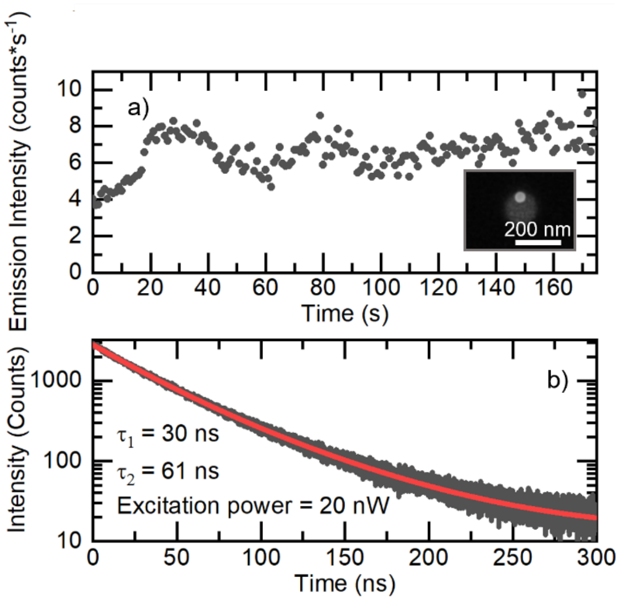


**Figure S7 –** *Additional optical characterization of the single NC in figure 3 of the main text a) The intensity-time trace measured over 180 s shows flickering of the NC between states on a millisecond time scale. b) The lifetime decay can be fitted (red dashed line) with two exponentially decaying functions yielding mean lifetimes of* $\tau_1$ *= 30 ns and* $\tau_2$ *= 61 ns.*

## 5. Saturation intensity curve

Figure S8 shows a refined measurement of the saturation excitation intensity for a SPE with g(2)(t = 0) = 0.29. The saturation curve ranges from 0 to 50 nW (117 W/cm$^2$) in 2.5 nW steps. The fit confirms the saturation intensity to be $I_s$ = 54 W/cm$^2$.

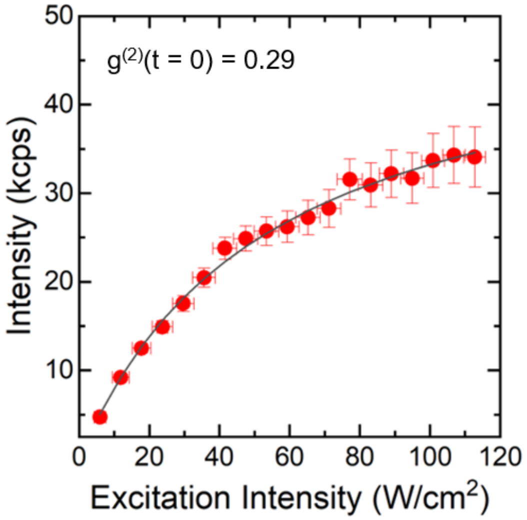


**Figure S8** – *Saturation curve (dots) and fit (blue line) of a SPE (g(2)(t = 0) = 0.29) with saturation intensity* $I_s$ *= 54 W/cm*$^2$.

## 6. Comparative emission spectra of SPE with different NCs in the nanohole

To identify the nature of the PL, an emission spectrum from nanoholes #1 and #3 was measured and fitted with a Lorentzian function yielding FWHM of 90 and 100 meV, respectively (Figure S9). This confirms that both SPEs originate from the decay of a single two-level system, most likely the exciton, regardless of the number of NCs present in the nanohole.

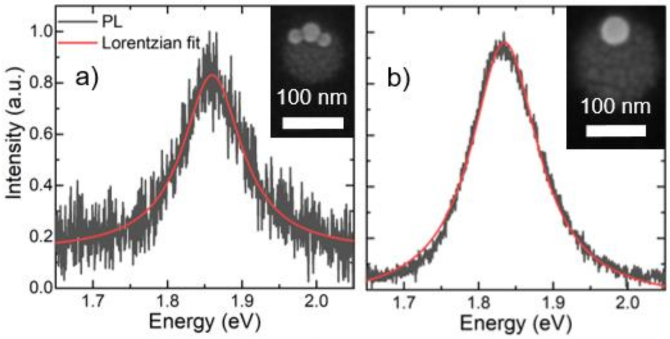


**Figure S9** – *Emission spectra (grey) and Lorentzian fit (red) of SPEs from holes #1 and #3 from Figure 4 in the main text containing 3 NCs and 1 NC, respectively (corresponding SEM in inset).*

## 7. Comparative study of NCs with and without silica coating

Figure S10 shows the PL lifetime decay from Silica coated and bare CdSe/CdS NCs. A double exponential fit retrieves lifetimes of $\tau_1$ = 53 ns and $\tau_2$ = 236 ns before coating and $\tau_1$ = 59 ns and $\tau_2$ = 258 ns after coating. The change in lifetime may be explain by the change in refractive index induced by the silica shell.

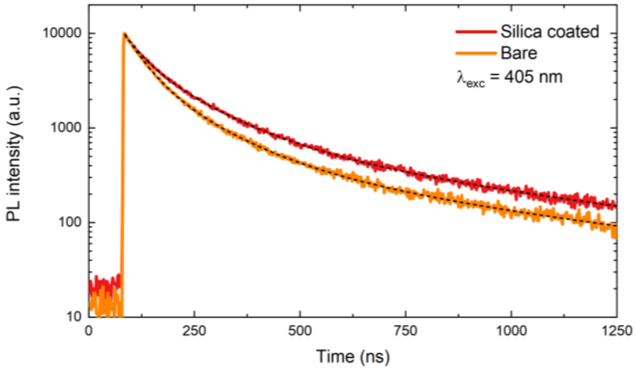


**Figure S10** – *Emission lifetime of the NCs before (orange curve) and after silica coating (red curve) collected from solution. The PL decays have been collected at the emission peak (648 nm for silica coated NCs and 645 nm for bare ones).*

## 8. Table of all the g(2)(t=0) measurements values acquired as a function of the number of NCs filling a hole

| 1 NC | 2 NCs | 3 NCs | 4 NCs | 5 NCs |
|---|---|---|---|---|
| 0.37 | 0.63 | 0.59 | 0.69 | 0.87 |
| 0.33 | 0.29 | 0.34 | 0.58 | 0.83 |
| | 0.43 | 0.43 | | 0.6 |
| | 0.47 | | | 0.68 |
| | 0.57 | | | 0.83 |
| | | | | 0.98 |
| | | | | 0.68 |
| | | | | 0.8 |
| | | | | 0.83 |
| | | | | 0.54 |
| | | | | 0.79 |
| | | | | 0.94 |
| | | | | 0.54 |
| | | | | 0.71 |
| | | | | 0.54 |
| | | | | 0.51 |
| | | | | 0.83 |
| | | | | 0.67 |

**Table S3** – *g(2(t=0)) values acquired as a function of the number of NCs filling a hole.*

## References


(1) Malaquin, L.; Kraus, T.; Schmid, H.; Delamarche, E.; Wolf, H. Controlled Particle Placement through Convective and Capillary Assembly. *Langmuir* **2007**, *23*, 11513–11521.

(2) Ni, S.; Leemann, J.; Wolf, H.; Isa, L. Insights into Mechanisms of Capillary Assembly. *Faraday Discuss.* **2015**, *181*, 225–242.

(3) Ni, S.; Isa, L.; Wolf, H. Capillary Assembly as a Tool for the Heterogeneous Integration of Micro- and Nanoscale Objects. *Soft Matter* **2018**, *14*, 2978–2995.

(4) Chen, R.; Zhang, L.; Zang, D.; Shen, W. Wetting and Drying of Colloidal Droplets: Physics and Pattern Formation. In *Advances in Colloid Science*; Muzibur Rahman, M., Mohamed Asiri, A., Eds.; IntechOpen, 2016.

(5) Parsa, M.; Harmand, S.; Sefiane, K. Mechanisms of Pattern Formation from Dried Sessile Drops. *Adv. Colloid Interface Sci.* **2018**, *254*, 22–47.
